# Reconstruction with double pedicel fibular graft and ankle arthrodesis for aggressive chondroblastoma in the distal tibia

**DOI:** 10.1186/s12957-016-0839-z

**Published:** 2016-05-12

**Authors:** Jian Fan, Shan-zhu Li, Jiong Mei, Guang-rong Yu

**Affiliations:** Department of Orthopedics, TongJi Hospital, TongJi University, 389 XinCun road, Shanghai, China

**Keywords:** Ankle arthrodesis, Aggressive, Chondroblastoma, Distal tibia

## Abstract

**Background:**

Aggressive chondroblastoma of the distal tibia is rare, and below-knee amputation had been the standard surgical procedure.

**Case presentation:**

We reported an additional case and reviewed the existing literature. A 20-year-old man with a 2-month history of right ankle pain and swelling underwent distal tibia wide resection, double pedicle fibular, autogenous iliac bone graft, and ankle arthrodesis. He had no pain, no limitation in daily activities, and no evidence of local recurrence and infection; the Musculoskeletal Tumour Society Score (MSTS) is 86 % at the final follow-up.

**Conclusions:**

Double pedicel fibular graft and ankle arthrodesis may be an effective and economical alternative method for aggressive chondroblastoma in the distal tibia.

**Electronic supplementary material:**

The online version of this article (doi:10.1186/s12957-016-0839-z) contains supplementary material, which is available to authorized users.

## Background

Chondroblastoma of bone, considered a benign lesion, is an uncommon condition that has a predilection for secondary ossification centers, particularly of the proximal humeral, tibia, and distal femur [1]. It occurs predominantly in the second decade, more commonly in males. At times, chondroblastoma may be aggressive in character. Primary malignant form as well as malignant alteration in the form of chondrosarcoma has been reported in less than 10 % of cases [2]. The ovoid lesion with thin sclerotic margin, located centrally in the epiphysis expands rapidly, and its boundaries with the sponge bone are unclear. An adequate and rapid diagnosis, including histological verification, is necessary before definitive treatment. Radical surgical resection including the tract of previous biopsy or surgery, avoiding any adjuvant therapy, is indicated for aggressive tumors [3].

Aggressive chondroblastoma of the distal tibia is further rare. For patients with aggressive chondroblastoma, treatments including below-knee amputation and arthrodesis have been recommended. With the development of limb salvage techniques, arthrodesis is becoming preferred. Among the options of arthrodesis, vascularized free fibular and non-vascularized autogenous fibular grafts were regarded as the most chosen reconstruction according to situations. In this report, we describe the successful wide resection of aggressive chondroblastoma and reconstruction with double pedicel fibular graft and ankle arthrodesis.

## Case presentation

**Fig. 1:**
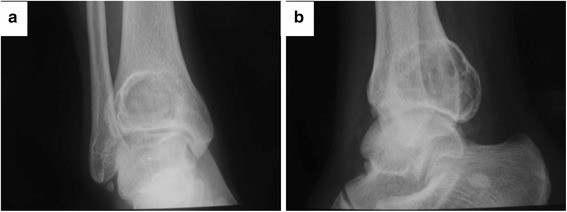
Pre-operative X-ray shows the lesion located in the distal of the right tibia. **a** Anteroposterior X-ray. **b** Lateral X-ray

**Fig. 2:**
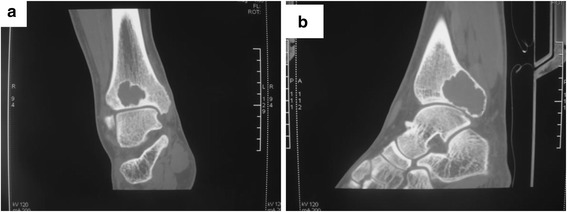
Transection scan of CT shows that the cortex of the distal tibia was destroyed by the tumor; however, fortunately, the medial malleolus was intact, and thus it gives us a chance to keep it. **a** Anteroposterior CT. **b** Lateral CT

**Fig. 3:**
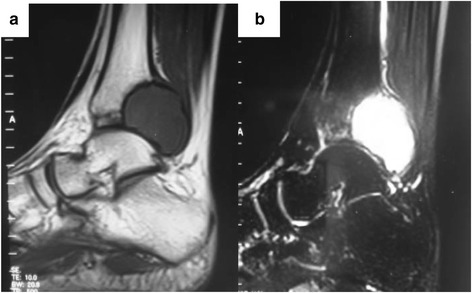
Pre-operative MRI shows that T1 low signal and T2 high signal of the lesion located in the distal of the right tibia protruded into the soft tissue. **a** T1 MRI. **b** T2 MR

**Fig. 4:**
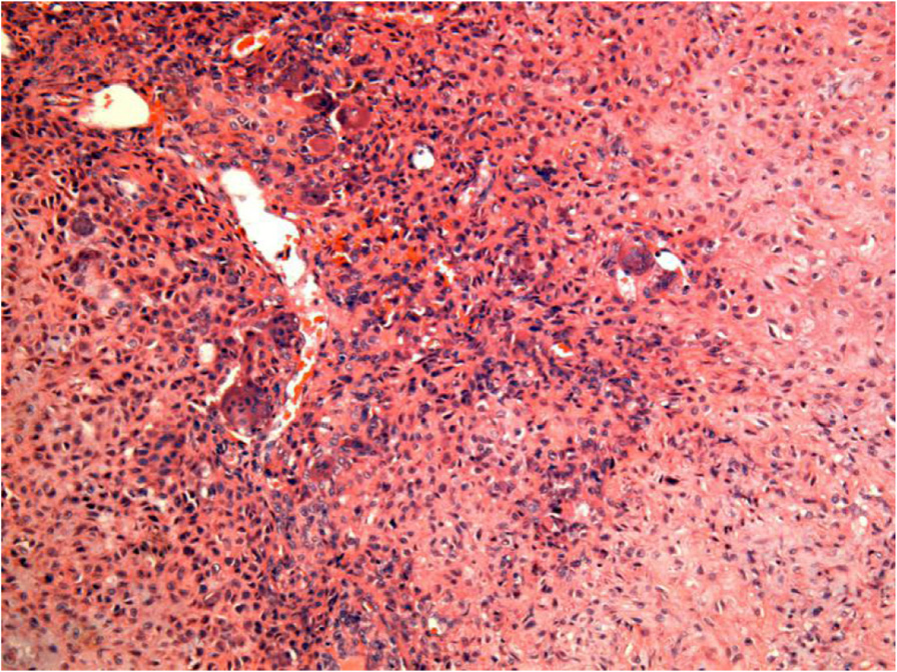
Microscopic image of aggressive Chondroblastoma showing the highly cellular tissue, variably differentiated and with discrete granulated to meshy calcification of the matrix and large multinuclear cells present in 20 % of cases. Magnification (×100) HE

In April 2010, a 20-year-old man with a 2-month history of right ankle pain and swelling presented in our hospital. His medical history was otherwise unremarkable. On physical examination, there was a mass with associated tenderness at the posterolateral aspect of the distal tibia. There was 18° restriction of plantar flexion and a 5° restriction of dorsiflexion. Plain radiograph and CT showed lytic lesions on posterolateral aspects of the distal tibia, and it appears as an ovoid lesion located centrally in the epiphysis (Figs. 1 and 2). MRI showed that the osteological area expands and its boundaries are unclear. The tumor mass expands beyond the lateral ankle and article surface of the distal tibia and protrudes into the soft tissue, but with the reservation of the fibula (Fig. 3). An incisional biopsy revealed aggressive chondroblastoma with an aneurysmal bone cyst. Microscopic fields in the tumor region showed highly cellular tissue, variably differentiated and with discrete granulated to meshy calcification of the matrix and large multinuclear cells present in 20 % of cases. The cyst fields were characterized by blood-filled cavities (Fig. 4). The patient also had a bone scan, chest radiograph, and CT of the lungs to determine if there are other metastases.

Posterolateral incisions were taken according to the site of biopsy and the position of the tumor. During the decomposition, the superficial peroneal nerve was identified and was retracted laterally. The flexor muscle of the thumb was exposed from peroneal muscle gap, and the muscle fibers were cut to the flexor muscle of the thumb, and, the remaining fibula periosteum and some muscle fibers were separated in the fibula. In the separation process, resection of the distal fibular of 12 cm in length should be performed and proximal vascular ligation should be also retained to form a distal pedicel fibula bone flap and peroneal artery including its branch vessels.

**Fig. 5:**
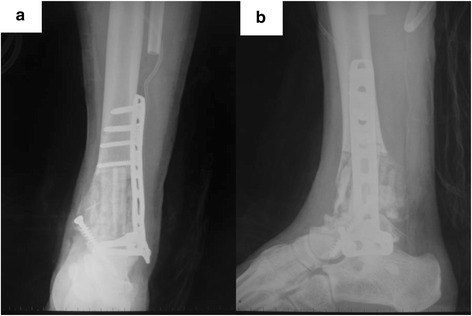
Post-operative X-ray shows the reconstruction of the distal tibia defect. **a** AP X-ray. **b** Lateral X-ray

**Fig. 6:**
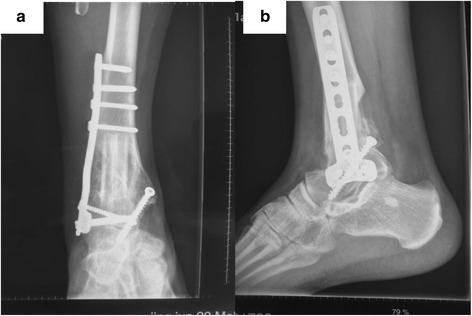
X-ray, 28 months after operation, shows the hypertrophy of the fibula and union of the junctions of the tibia-fibula and the fibula-talus. **a** AP X-ray. **b** lateral X-ray

After opening the extensor retinaculum, the joint capsule was exposed and meticulous dissection was carried out to preserve a wide protective margin of the tissue. Previous biopsy tracts were incorporated into the incision and completely excised with the specimen. According to the tumor position determined by CT, the posterolateral and central parts of the distal tibia were resected from 1 cm proximal along with the tumor remaining medial part. The soft tissue protruded by the tumor was also wide resected. After the fibula periosteum and the muscle fibers were separated at the fibular head, the fibula head was resected to maintain long enough pedicel and articular cartilage of the talus and medial ankle were eradicated while preparing two holes in the talus head. After distal fibular was folded up into two, the two pedicel graft were inserted between the medullary canal of the tibia and the holes in the talus. Then, a titanium plate between the distal tibia and talus was fixed. Attention should be taken to place the ankle in 5^0^–10^0^ valgus, 10^0^ external rotation, and a neutral dorsiflexion. An autogenous iliac bone graft was also inserted to fill the remaining defect in the tibia and recipient bed of the talus (Fig. 5). The patient’s ability to walk depended on radiographic evidence of bony healing. In general, the patient was kept non-weight bearing for 3 months, then progressed to full-weight bearing wearing a walking boot by 3 months, and was allowed to bear without wearing a boot beginning from 6 to 9 months. After that time, the patient was able to walk independently without pain. The patients is now in the third year of follow-up and continues to be free of pain and has had no further complains; plain radiographs show a solid bony union (Fig. 6).

### Discussion

Since chondroblastoma is a benign lesion wholly, curettage and osteoplasty using auto- and homograft are the main method for chondroblastoma in most cases and additional implants may be used if there is mechanical instability. For particularly aggressive tumors, marginal or wide resection is necessary. In exceptional cases, amputation may be necessary when the soft tissue and neurovascular bundle are extensively involved [4].

Tumors of the distal tibia are quite rare. For malignant and potential malignant tumors, below-knee amputation had been the standard surgical procedure [5] because of the limitation of soft-tissue coverage and satisfactory function of below-knee prosthesis [6]. Recently, social reasons and improved technique have made limb salvage in the distal tibia increasingly possible [7]. Among the many options of reconstruction, ankle endoprosthetic is not accepted by most surgeons because of its high rate of complications [8]. Arthrodesis was preferred, providing excellent stability of the ankle and avoiding problem relating to prosthetic implantation. Arthrodesis with auto-graft is an economical, safe, and effective method without complication of the rejection reaction and non-union, infection, osteolysis, fracture which carried by allograft. Compared with non-vascularized autogenous fibular grafts, vascularized fibula was also superior to non-vascularized grafts with reduced time to union and faster hypertrophy [9]. Casadei reported good functional and oncological results in 12 patients with malignant bone tumors of the distal tibia, treated by resection and arthrodesis with vascularized free autogenous bone graft [10]. Bishop et al. also succeed in reconstruction using a vascularized free fibular graft for the treatment of malignant tumors in the distal tibia [11]. But in their cases, mobility of the donor site and the complicated surgical procedure are a problem. Our retrospective case presents a different method of limb salvage in the treatment of distal tibia tumors. Same as the other methods, wide resection is necessary and the resection needs to include articular surface, ligaments, and the joint capsule. The local extent of the tumor also required resection of the most tibia to an extent that was incompatible with reconstruction of the joint anatomy, so in the case, according to the tumor position, the medial malleolus remained to strengthen the stability. Compared with the Casadei’s technique, we had not used the proximal and middle fibular graft but the distal fibular which can obtain sufficient auto-graft of bone in the same incision. The distal fibular graft can get long enough pedicle after resecting the fibular head and inserted between the tibia canal and talus which can provide an effective foundation of stability after doubling up. After that, autogenous iliac bone grafts were inserted to fill the remaining defect between the tibia and talus. This compound reconstruction in which procedure was simpler than the vascularized one was not the same totally with non-vascularized autogenous fibular grafts and had brought about sound fusion and minimum.

**Fig. 7:**
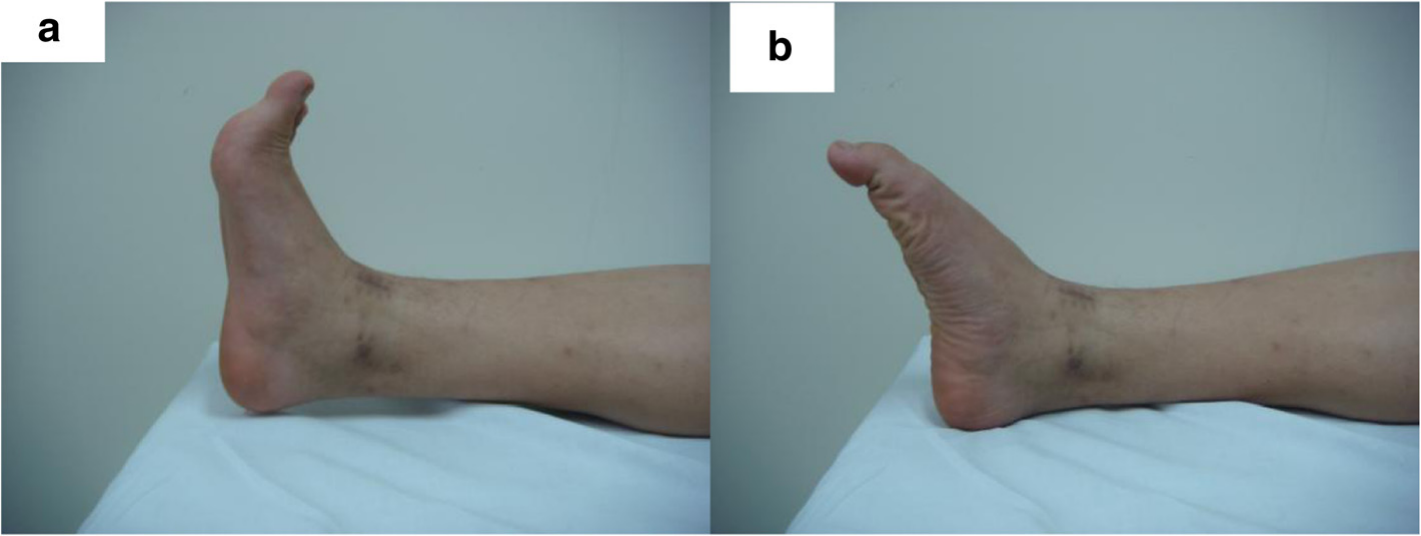
20° of tibiopedal motion. **a** Plantar flexion of tibiopedal. **b** Dorsiflexion of tibiopedal

We used a plate and a screw as the fixation device in this patient because of enough soft tissue. This procedure was also simpler than intramedullary nailing suggested by Moore’s series for shortage of the soft tissue. In our experience, wide resection and reconstruction with double pedicel fibular graft and ankle arthrodesis is a valuable alternative in limb salvage for aggressive chondroblastoma in the distal tibia. This technique avoids the donor site morbidity of auto-graft used, the talus collapse and bone loss associated with an ankle endoprosthesis. After ankle arthrodesis, our patient had 20° of tibiopedal motion (Fig. 7) and no complications of mal-union, pseudarthrosis, loss of internal fixation, post-operative infection, skin necrosis, and chronic edema at 4 years of follow-up. There was no shortening of the lower extremity, and their gait was nearly normal (Additional file 1).

Degenerative arthrosis may develop in the sub-talus joint after ankle arthrodesis [12]. through there was no evidence of arthrosis in the sub-talus or other joints of the foot in our patient; further longer follow-up clinical effect should take in next step.

## Conclusions

We describe a novel technique of double pedicel fibular graft and ankle arthrodesis for aggressive chondroblastoma in the distal tibia. This technique allowed for retention of protective motor function and avoidance of ankle immobilization, while providing no pain, no limitation in daily activities, and no evidence of local recurrence and infection, 86 % of MSTS score, and patient satisfaction compared with traditional below-knee prosthesis after amputation.

## Consent

Written informed consent was obtained from the patient for publication of this manuscript and any accompanying images. A copy of the written consent is available for review by the Editor-in-Chief of this journal.

## Additional file

Additional file 1: The gait was nearly normal on 28 months after operation. (MPEG 10400 kb)
